# New Insight into the Evolution of Symbiotic Genes in Black Locust*-*Associated Rhizobia

**DOI:** 10.1093/gbe/evz116

**Published:** 2019-06-13

**Authors:** Zhenshan Liu, Weimin Chen, Shuo Jiao, Xinye Wang, Miaochun Fan, Entao Wang, Gehong Wei

**Affiliations:** 1 State Key Laboratory of Crop Stress Biology in Arid Areas, Shaanxi Key Laboratory of Agricultural and Environmental Microbiology, College of Life Sciences, Northwest A&F University, Yangling, Shaanxi, China; 2 Departamento de Microbiología, Escuela Nacional de Ciencias Biológicas, Instituto Politécnico Nacional, México, D.F., Mexico

**Keywords:** symbiotic genes, phylogeographic pattern, genetic structure, approximate Bayesian computation

## Abstract

Nitrogen fixation in legumes occurs via symbiosis with rhizobia. This process involves packages of symbiotic genes on mobile genetic elements that are readily transferred within or between rhizobial species, furnishing the recipient with the ability to interact with plant hosts. However, it remains elusive whether plant host migration has played a role in shaping the current distribution of genetic variation in symbiotic genes. Herein, we examined the genetic structure and phylogeographic pattern of symbiotic genes in 286 symbiotic strains of *Mesorhizobium* nodulating black locust (*Robinia pseudoacacia*), a cross-continental invasive legume species that is native to North America. We conducted detailed phylogeographic analysis and approximate Bayesian computation to unravel the complex demographic history of five key symbiotic genes. The sequencing results indicate an origin of symbiotic genes in Germany rather than North America. Our findings provide strong evidence of prehistoric lineage splitting and spatial expansion events resulting in multiple radiations of descendent clones from founding sequence types worldwide. Estimates of the timescale of divergence in North American and Chinese subclades suggest that black locust-specific symbiotic genes have been present in these continent many thousands of years before recent migration of plant host. Although numerous crop plants, including legumes, have found their centers of origin as centers of evolution and diversity, the number of legume-specific symbiotic genes with a known geographic origin is limited. This work sheds light on the coevolution of legumes and rhizobia.

## Introduction

Rhizobia are unique among soil bacteria for their ability to perform endosymbiotic nitrogen fixation with legumes in a specialized structure known as the nodule (Masson-Boivin et al. 2009). A successful symbiotic interaction between rhizobia and their compatible host involves genes essential for nodulation (*nod/nol/noe*) and nitrogen fixation (*nif/fix/fdx*) (Masson-Boivin et al. 2009; Oldroyd et al. 2011). Initiation of symbiosis requires a complicated molecular dialogue between the two partners (Fisher and Long 1992). Plant flavonoids activate the expression of *nod* genes in rhizobia that encode enzymes responsible for the synthesis of lipochito-oligosaccharidic Nod factors, while these bacteria-derived signaling molecules in turn trigger nodule morphogenesis and play a key role in defining host range and specificity (Perret et al. 2000; Wang et al. 2012; Ling et al. 2016). The *nif/fix/fdx* genes are required for nitrogen fixation following plant infiltration. These genes are generally clustered in symbiotic modules on mobile genetic elements such as symbiotic plasmids (Galibert et al. 2001) and distributed across taxa via repeated and independent horizontal gene transfer (HGT), a process by which genetically unrelated organisms exchange genes that can promote rapid adaptation (Gibson et al. 2008; Oldroyd et al. 2011; Remigi et al. 2016). The ancestors of extant rhizobia are assumed to have acquired and improved symbiotic capacities under host selection much more recently (Turner and Young 2000; Lavin et al. 2005; Sprent and James 2007). In the context of HGT, evolutionary histories and selective forces operating on symbiotic genes may differ from those of housekeeping genes not involved in symbiosis (Remigi et al. 2016).

Extensive genetic studies based on housekeeping genes are indicative of emerging trends in rhizobial biogeography. Specifically, the distribution patterns of rhizobial species have been strongly influenced by edaphic and climatic factors and host distribution, and the population genetic structure of rhizobial species is qualitatively characterized by a pattern of isolation by distance (Eardly and Xu 2010; Zhang et al. 2011; Tian et al. 2012). It is widely accepted that the geographic distribution patterns of symbiotic rhizobia are pivotal to the ecological success of legume plants (Parker et al. 2006). However, recent research demonstrated that legumes have stringently selected the symbiotic genotype, but not the genomic background of rhizobia (Parker 2012; Li et al. 2016). This is evident from the fact that colonization of a novel environment by a legume and its symbionts with weak local adaptation may lead to out-competition of invasive rhizobial partners by native nonsymbiotic rhizobia that acquire symbiotic genes (Sullivan et al. 1995; Nandasena et al. 2006). Further information on plant-rhizobia coevolution should be obtained from symbiotic genes, rather than from the rhizobial species that now bear them. Thus, unraveling the evolutionary history and biogeographic structuring of rhizobial symbiotic genotypes could make it possible to gain meaningful insight into the dynamic adaptive evolution of rhizobial symbiotic compatibility with legume hosts and specific environments, which is a precondition in particular for the agricultural application of elite rhizobial inoculants.

Black locust (*Robinia pseudoacacia*) is one of the most important cross-continental dispersal legumes for restoration efforts across the world. Black locust is believed to have originated in North America, been transported to Europe in the 17th century, and then spread to Asia in the last century (Sabo 2000; Ren et al. 2014). Previous studies on symbiotic diversity in black locust have shown that in its native environment and in Europe and Asia, this plant is nodulated by various rhizobial genera including *Rhizobium*, *Sinorhizobium*, and *Mesorhizobium*, of which *Mesorhizobium* are dominant in root nodules (Wei et al. 2009; Mierzwa et al. 2010). Despite taxonomic diversity, black locust*-*associated rhizobia from different continents share highly similar *nod* and *nif* genes, suggesting that these symbiotic genes might have a monophyletic origin and have conferred the symbiotic ability to previously nonsymbiotic lineages of rhizobia. However, limited bacterial sampling provides incomplete information about the historical microevolutionary forces driving the evolution of legume-rhizobia symbiosis. Additional efforts are necessary to discern whether the migration of plant hosts has facilitated HGT across large geographic distances, to assess its influence on the current spatial genetic structure of symbiotic genes on a worldwide scale, and perhaps most importantly, to investigate the geographic origin and evolutionary history of symbiotic genes.

Powerful approaches based on coalescent theory for determining demographic and evolutionary histories are now available (Wakeley 2008; Grünwald and Goss 2011). Bayesian phylogeography makes use of geographic information to provide model-based inference of geographic locations of ancestral lineages while accommodating phylogenetic uncertainty (Vaughan et al. 2014). Approximate Bayesian computation (ABC) takes advantage of coalescent simulations and likelihood-free inference to contrast complex demographic scenarios that incorporate most evolutionary processes such as migration, recombination, population divergence, and changes in population size (Bertorelle et al. 2010). IMa2 is a Bayesian- and coalescent-based method that estimates the divergence time (*t*) of a single ancestral population with an effective population size (*θ*_A_) split into two derived populations that may have asymmetrical migration rates (*m*_1_ and *m*_2_) and different sizes (*θ*_1_ and *θ*_2_) (Hey 2010). Other Bayesian clustering methods such as STRUCTURE (Evanno et al. 2005) and BAPS (Corander et al. 2013) assign individuals into underlying groups that may not correspond to geographically defined populations, thus helping identify introductions, discover cryptic species, and define population structure without a priori knowledge of gene/genotype flow. The combined application of these methods could provide critical novel insight into the demographic history of organisms (Goss et al. 2014).

The objective of the present study was to examine the evolutionary past of symbiotic genes in rhizobia associated with black locust within the context of worldwide migration, and to test whether the proposed center of origin of black locust (North America) is also the center of origin of symbiotic genes using Bayesian phylogeography and ABC. We sampled key populations of *Mesorhizobium* from North America, Germany, and China to investigate the center of origin via genotyping of multiple loci.

## Materials and Methods

### Sampling and DNA Data Collection

The sampling of nodules aimed to incorporate filed populations spanning the current distribution of black locust in China. Nodules were also collected from one site in North America (Berkley, California) and one site in Germany (Freiburg). Sampled trees were at least 100 m apart. We obtained a total of 286 *Mesorhizobium* strains, comprising 227 Chinese strains, 48 German strains, and 11 North American strains (supplementary fig. S1 and table S1, Supplementary Material online). Genomic DNA was extracted using the CTAB method (Andreou 2013). Twelve gene fragments from all *Mesorhizobium* individuals were sequenced, including 16S rDNA (1,302 bp) for species identification, five symbiotic genes located in the *nod-nif* cluster, namely *nodA* (535 bp), *nodC* (798 bp), *nifA* (693 bp), *nifH* (686 bp), and *nolT* (596 bp), and six housekeeping genes, namely *recA* (475 bp), *atpD* (468 bp), *glnII* (658 bp), *dnaJ* (758 bp), *rpoA* (684 bp), and *gryB* (713 bp). All these regions were sequenced using in-house primers and all loci were aligned in MEGA 7.0 (Kumar et al. 2016).

### Analysis of Sequence Diversity and Population Structure Based on Housekeeping and Symbiotic Genes

Nucleotide diversity and neutrality tests for each locus were performed using START 2 (Jolley et al. 2001) and ARLEQUIN (Excoffier and Lischer 2010). We also applied the relative abundance of nonsynonymous and synonymous polymorphisms (*p*_N_ and *p*_S_) furthermore to measure the direct effect of natural selection in coding sequences (Stukenbrock and Bataillon 2012). The formatted input files for STRUCTURE (Evanno et al. 2005) were generated using xmfa2struct (available at http://www.xavierdidelot.xtreemhost.com/clonalframe.htm) based on two concatenated sequence data sets. The linkage model was run assuming the number of clusters *K *=* *2–10 with a burnin of 100,000 iterations and 400,000 further iterations. STRUCTURE results were postprocessed using CLUMPAK (Kopelman et al. 2015), in which Δ*K* statistics were applied to infer the most likely number of genetic clades (Kopelman et al. 2015). The relationships among the haplotypes of symbiotic genes were depicted in a genealogical tree generated using ClonalFrame and a minimum spanning network constructed using the R package poppr (Kamvar et al. 2014).

### Recombination and Linkage Disequilibrium Test

A coalescent-based approach implemented in ClonalFrame was applied to estimate the relative probabilities that a nucleotide is changed as a result of recombination versus mutation. The importance of recombination for all five symbiotic loci was estimated via the homoplasy test, which examines the probability of a single site change occurring more than once during the evolutionary history of these sequences. The homoplasy index, which ranges from 0 (for a clonal population) to 1 (for a population under free recombination) (Maynard and Smith 1998), was calculated for each symbiotic gene with PAUP* (available at: http://phylosolutions.com/paup-test/). The linkage disequilibrium for symbiotic genes was assessed by evaluating the observed linkage among loci against the expected distributions from permutation using the index of association (*I*_A_), and departure from the null hypothesis (no linkage disequilibrium, *I*_A_ = 0) was assessed with 1,000 permutations using clone-corrected data. We also examined the *I*_A_ of each sampling site with strain numbers >9 as well as within each STRUCTURE genetic clade of symbiotic genes.

### Determining the Demographic History of Symbiotic Genes

Recent fluctuations in population size were assessed by calculating common summary statistics (Tajima’s *D* and Fu’s *F*_s_) for the entire symbiotic gene data set and each STRUCTURE clade separately using Arlequin (Excoffier and Lischer 2010). Mismatch distributions (Harpending 1994), the frequency distributions of numbers of mismatches between pairwise sequences, were compared with expected distributions obtained under the demographic scenarios of stable population size, sudden population expansion, and spatial expansion for evidence of past spatial or demographic expansions. Goodness-of-fit between observed and simulated distributions was estimated by calculating the sum of squared deviations (*SSD*) of the observed data relative to the model, and Harpending’s raggedness statistic (*H*_Rag_) (Harpending 1994), a measure of the irregularity of the shape of the observed distribution. Confidence intervals (CIs) for mismatch distribution parameters were obtained by performing 1,000 bootstrap replicates using Arlequin (Excoffier and Lischer 2010). Changes in population size were also validated for each STRUCTURE clade using the coalescent-based method in LAMARC (Kuhner 2006).

### Identifying the Phylogeographic Root of Symbiotic Genes

We performed a Bayesian structured coalescent phylogeographic analysis to infer the most likely clade/geographic origin (“root state”) and movement of symbiotic genes using MultitypeTree (Vaughan et al. 2014) implemented in BEAST (Bouckaert et al. 2014). This method uses the locations or states of samples as prior knowledge to reconstruct ancestral states of tree nodes, including estimation of the root state posterior probability. To initialize BEAST, jModelTest (Darriba et al. 2012) and the Akaike information criterion were used to obtain the appropriate nucleotide substitution models, favoring a JC model (Jukes and Cantor 1969) for each alignment. Comparison of different molecular clocks for each symbiotic gene suggested that four of five loci fit a strict clock model, where the UCLD.stdev parameter (the SD of the uncorrelated lognormal relaxed molecular clock) indicated no rate heterogeneity among branches within the data. In contrast, the *nifH* gene exhibited high variation in rate among branches and should fit a relaxed lognormal molecular clock model (supplementary table S2, Supplementary Material online). To ensure adequate sampling of parameters, we conducted five independent BEAST analyses with MCMC simulations of 200 million iterations for each locus, for all data, and for Clade III only which comprised three geographic subclades, with sampling every 1,000 iterations after 25% burn-in. Multilocus analyses were also run for all data and for Clade III only. Data were analyzed under a strict clock rate, with effective sample size estimates typically >200, and parameter trace plots exhibiting MCMC convergence and good mixing in each independent run. Maximum clade credibility trees were summarized from 7,500 trees remaining after the removal of burn-in with TreeAnnotator.

### Estimation of Divergence Times

IMa2 analyses were conducted to estimate the divergence time between each pair of STRUCTURE clades and between subclade pairs in Clade III with all symbiotic loci combined (Hey 2010). For each locus, we opted for the infinite sites substitution model and ran the program three times to ensure convergence under the Metropolis-Hastings MCMC algorithm. For each run, we used a geometric heating model with 80 chains of one million generations beyond a 100,000 generations burn-in, with geometrical increment parameters of 0.97 and 0.3. After exploratory running, we set the maxima for uniform prior distributions of the parameters as follows: migration rate (*m*) = 1.0, population size (*q*) = 20.0, and divergence time (*t*) = 50.0.

Since all parameters estimated from IMa2 are scaled by mutation rate (e.g., divergence time *t* = *T*μ, where *T* is the time in generations or years and μ is the mutation rate), and no useful fossils are available for bacteria to calibrate the estimation of demographic parameters, we used the mutation rate estimated from ABC analysis in the present study (as detailed in the following section) to convert the scaled estimation to more biologically meaningful units.

### Selecting Evolutionary Models of Symbiotic Genes

To better understand the evolution of symbiotic genes, we evaluated alternative evolution scenarios using the ABC approach implemented in ABCtoolbox (Samuel et al. 2010), a program that facilitates the integration of summary statistics calculation, simulations, parameter estimation, and validation. We compared 19 competing evolutionary models (supplementary fig. S2, Supplementary Material online), representing all possible evolutionary relationships among clades. To simplify the computation, we assumed that the effective size of all populations remained stable over time until instantaneous population splitting events. Models were defined using 9 − 13 historical parameters for which we assumed prior distributions with wide bounds to reflect the uncertainty associated with the limited available information on the evolution of symbiotic genes (supplementary table S3, Supplementary Material online).

Summary statistics characterizing genetic diversity within clades and genetic differentiation among clades were assumed to be the most appropriate to allow discriminating among the compared hypotheses. A total of 21 summary statistics (supplementary fig. S3, Supplementary Material online) that describe patterns of variation within and between clades were calculated using ARLSUMSTAT (Excoffier and Lischer 2010). For each model, we ran one million exploratory simulations under the coalescent model with demographic parameter values drawn from their prior distributions at random. The values simulated under each scenario were then compared with the summary statistics from the observed data set by computing the Euclidean distance. The posterior probability of each competing scenario was estimated by applying generalized linear model (Leuenberger et al. 2010) regression on the 0.5% of simulated data sets closest to the observed data set, and Bayes factors for each pairwise comparison between scenarios were calculated as the ratio of the marginal densities. The alternative hypothesis can be rejected if the Bayes factor between two scenarios was >3.

Validation of model choice was conducted first by comparing the marginal density of the observed data with marginal densities obtained from the retained simulations to compute a *P* value to determine whether models could faithfully reproduce the observed data. We assumed that *P* values <0.05 indicate that few of the simulated data sets have marginal density that is smaller than or equal to that of the observed summary statistics, and such models were excluded from the final model choice procedure. We subsequently estimated type I and type II errors for the remaining models after filtering and removing poorly fitting models to estimate the ability to distinguish between models. Briefly, for each demographic model, we simulated 1,000 pseudo-observed data sets (PODs) under each model and analyzed them using our ABC procedure for model choice. Each POD was treated as observed data and used to calculate the posterior probability of each scenario. We estimated type I error as the proportion of PODs for which the correct scenario did not display the highest posterior probability. We then empirically estimated the type II error by computing the proportion of pods erroneously assigned to a given scenario.

We evaluated whether the selected models were good approximations of true scenarios by generating a density distribution for each statistic and calculating the 2.5 and 97.5 percentiles of the distribution. Validation of the estimated parameters was performed by examining the coverage properties of the posterior distribution as described previously (Wegmann et al. 2009). We obtained 1,000 PODs with known parameter values from the simulations themselves, and checked the distribution of the posterior quantiles for uniformity using Kolmogorov–Smirnov tests. We employed the best model to estimate the divergence time between Chinese and North American subclades in Clade III (i.e., Model 20 in supplementary fig. S4, Supplementary Material online).

## Results

### Sequence Diversity, Recombination, and Genetic Structure

Analysis of nucleotide diversity in the 286 *Mesorhizobium* strains revealed sequence variation present across all six housekeeping loci and five symbiotic loci. The average nucleotide diversity was an order of magnitude lower for symbiotic loci than for housekeeping loci (table 1). All loci exhibited a statistically nonsignificant departure from equilibrium conditions using both Tajima’s *D* and Fu’s *F*_s_ values. Additionally, these loci showed a higher rate of synonymous substitutions than nonsynonymous substitutions, with both *d*_N_/*d*_S_ and *p*_N_/*p*_S_ ratios well <1, and *p*_N_/*p*_S_ ≈ *d*_N_/*d*_S_, indicating that sequence variation was largely neutral (see Discussion for further interpretation). The concatenated sequences defined 156 haplotypes for housekeeping genes and 74 for symbiotic genes. According to estimates for *ρ*/*θ* (the ratio of rates at which nucleotides become substituted due to recombination and mutation) and *r*/*m* (the relative contribution of recombination and mutation in the generation of genetic diversity), the rate of recombination was clearly lower than mutation in both data sets, yet recombination played a more important role in generating the genetic variation in housekeeping genes compared with symbiotic genes. However, clonality was detected in both data sets, and the *I*_A_ values calculated for both clone-corrected data sets provides strong evidence for the hypothesis of clonal reproduction (Housekeeping genes: *I*_A_=54.9, Symbiotic genes *I*_A_=5.44, both *P *<* *0.001), although the possibility that limited migration between geographically isolated populations might contribute to linkage disequilibrium in the face of frequent local recombination cannot be excluded. In order to determine whether frequent mixis of genotypes within population occurred, we examined the *I*_A_ of each sampling site with strain numbers >9 as well as China as a whole. We observed significant linkage disequilibrium at scales of single site and throughout China (supplementary fig. S5, Supplementary Material online). We did not observed a much greater amount of disequilibrium among strains collected throughout China than from one area of China, rejecting the hypothesis of restricted migration between populations. Moreover, the homoplasy index for all symbiotic genes showed a weak trend of departure from zero. We therefore concluded that strong recombination was largely absent within these five symbiotic genes.

**Table 1:** MLST Sequence Diversity and Neutrality Test of Housekeeping and Symbiotic Genes in the 286 *Mesorhizobium* Strains

Locus	Alleles	*p*_N_/*p*_S_	*d*_N_/*ds*	*Pi *×* *10^−3^	*θ*_w_×10^−3^	Fu’s *F*_s_ (*P* value)	Tajima’s *D* (*P* value)	*ρ*/*θ* (95% CI)	*r*/*m* (95% CI)	*H*
*atpD*	86	0.1407	0.116	74.76	62.75	−1.854 (0.447)	0.589 (0.789)	0.882 (0.608, 1.280)	3.62 (2.62, 5.06)	—
*DnaJ*	84	0.0444	0.038	58.70	52.08	1.647 (0.695)	0.381 (0.74)	0.572 (0.387, 0.883)	4.58 (3.42, 6.87)	—
*gln2*	70	0.1031	0.088	78.54	56.18	10.832 (0.906)	1.229 (0.917)	0.374 (0.237, 0.633)	4.35 (3.05, 6.02)	—
*gryB*	76	0.0863	0.074	66.67	56.07	5.48 (0.826)	0.607 (0.791)	0.519 (0.316, 0.727)	3.90 (2.80, 5.41)	—
*recA*	69	0.0195	0.017	50.03	51.94	−2.421 (0.386)	−0.248 (0.459)	0.387 (0.226, 0.666)	4.10 (2.84, 5.94)	—
*rpoA*	68	0.0329	0.029	40.37	40.42	−1.334 (0.494)	−0.004 (0.601)	0.487 (0.280, 0.764)	4.08 (2.75, 5.81)	—
*nifA*	17	0.3207	0.350	6.61	4.40	0.822 (0.669)	1.287 (0.906)	1.627 × 10^−4^ (3.482 × 10^−5^, 1.377 × 10^−2^)	0.43 (0.02, 15.58)	0.286
*nifH*	45	0.0646	0.065	13.15	14.27	−5.174 (0.173)	−0.23 (0.48)	0.026 (0.010, 0.089)	0.18 (0.06, 1.19)	0.425
*nodA*	7	0.7407	0.759	2.04	1.80	0.00655 (0.55)	0.257 (0.649)	3.092 × 10^−4^ (1.197 × 10^−4^, 3.036 × 10^−2^)	0.18 (0.01, 16.23)	0
*nodC*	8	0.1067	0.110	2.69	1.83	0.290 (0.61)	0.912 (0.851)	3.811 × 10^−2^ (8.977 × 10^−3^, 0.513)	0.18 (0.04, 2.55)	0.227
*nolT*	18	0.1253	0.126	5.88	5.12	−0.756 (0.464)	0.381 (0.71)	9.429 × 10^−5^ (3.210 × 10^−5^, 7.536 × 10^−3^)	0.40 (0.02, 14.32)	0.300

Note.—_N_, nonsynonymous polymorphisms; *p*_S_, synonymous polymorphisms; *d*_N_, nucleotide diversity in nonsynonymous sites; *d*_S_, nucleotide diversity in synonymous sites; *ρ*, recombination rate; *θ*, mutation rate; *ρ*/*θ*, recombination to mutation ratio; *r*/*m*, the ratio of probabilities that a site is altered through recombination or mutation; *H*, homoplasy index.

**Fig. 1.**
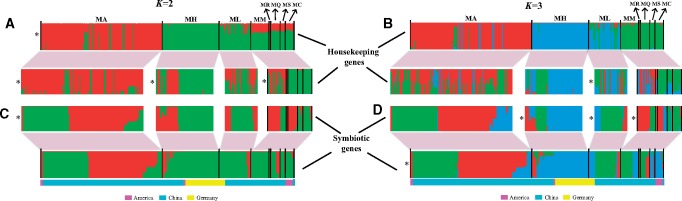
—Genetic structure of rhizobial populations based on housekeeping genes (*A* and *B*) and symbiotic genes (*C* and *D*) for all 286 *Mesorhizobium* strains associated with black locust and each species/species combination, assuming correlated allele frequencies and a linkage model with marker distances in base pairs. Each sample is depicted as a vertical bar colored by the ancestry proportion of genetic components inherited from *K *=* *2 (*A* and *C*) or *K *=* *3 (*B* and *D*) inferred ancestral gene pools. Asterisks indicate the resolution at the best *K* value based on the Δ*K* method. The best resolution at *K *=* *3 for the symbiotic genes (*D*) of all strains results in three main clades with a membership of >0.67 for each individual. Individuals are grouped according to species and geographical region as labeled above or below the figures. Species abbreviations are as follows: MA, *M. amorphae*; MH, *M. huakuii*; ML, *M. loti*; MM, *M. metallidurans*; MR, *M. ribiniae*; MQ, *M*. *qingshengii*; MS, *M. sangaii*; and MC, *M. ciceri*.

To better understand the role of horizontal transfer in shaping genetic structure, we compared patterns of genetic composition between housekeeping and symbiotic genes for the whole collection of *Mesorhizobium* individuals and each mesorhizobial species. Bayesian estimation of assignment probabilities for each individual showed a marked genetic break among species at *K *=* *2 or *K *=* *3 for housekeeping genes, although some individuals exhibited a relative low level of ancestry from their putative species (fig. 1). This was evident from a genealogical analysis of the derived housekeeping gene haplotypes with ClonalFrame (supplementary fig. S6, Supplementary Material online), a model-based approach that infers the clonal relationship of bacteria while accounting for recombination events. However, for symbiotic genes, we observed a substantial genetic admixture among species. In addition, each species/species combination, except for *M. huakuii* (MH), displayed a different subdivision pattern between housekeeping and symbiotic genes, indicating a considerable degree of HGT occurring among strains. Assignment analysis incorporating all individuals indicated that the most likely number of clades was three for symbiotic genes. Percentage membership (*q*) for the three genetic clades was calculated for each individual, with a threshold probability of *q *>* *0.67 required to assign an individual to one of the clades identified. Clade I (red) and Clade II (green) included 82 and 97 individuals (*q *>* *0.9), respectively. These two clades mainly consisted of Chinese individuals, with the exception of 2 North American individuals in Clade I. Clade III (blue) included all 48 individuals of German lineage, 9 individuals of North American lineage, and 31 individuals from China. The remaining 19 strains were highly admixed and difficult to assign to any clades using the same method. Restricted but asymmetric gene flow was apparent among clades and most gene flow involved Clade I and Clade III. In accordance with the results of Bayesian clustering analysis, ClonalFrame revealed well-separated clades as indicated by STRUCTURE results (supplementary fig. S6, Supplementary Material online).

**Fig. 2.**
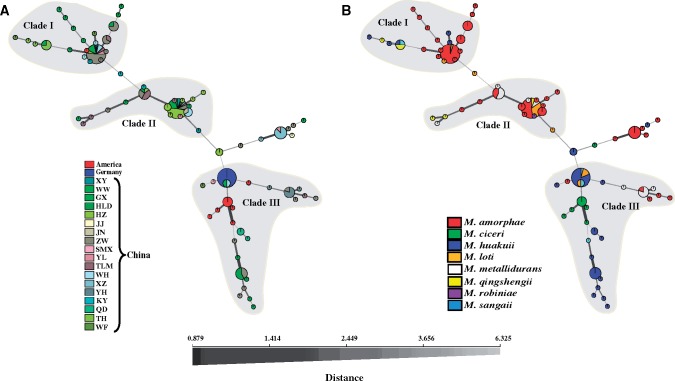
—Minimal spanning network displaying the relationship between multilocus haplotypes of symbiotic genes at continental (*A*) and species (*B*) levels. Each haplotype is represented by a node, the size of which is proportional to the number of clones recovered for that haplotype. The width and shading of edges indicate relatedness (thicker, darker edges represent close relatedness, while thin, pale edges represent greater distance). Edge length is arbitrary. Shaded haplotypes indicate corresponding clades in the structure.

**Fig. 3.**
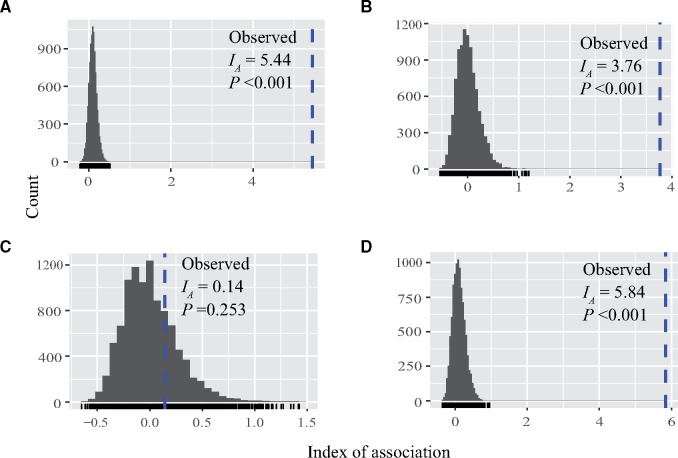
—Index of association (*I*_A_) test of recombination for all *Mesorhizobium* individuals and for each clade. Based on the frequency distribution of *I*_A_, the hypothesis of no linkage among markers is rejected (*P *<* *0.001) for all data (*A*), Clade I (*B*), and Clade III (*D*), indicating clonal populations. There is no evidence of linkage disequilibrium among loci (*P *=* *0.263) for Clade II (*C*), consistent with a sexual-like recombining clade.

The 74 haplotypes of symbiotic genes formed a unique path within a single minimal spanning network, which contained 95 informative single nucleotide polymorphisms (SNPs) that marked the evolutionary history of symbiotic genes plus 25 noninformative SNPs that were specific to single haplotype (fig. 2). Each STRUCTURE clade was represented by at least 17 haplotypes in the network and dominated by one most frequent haplotype that could be assigned as the putative ancestral or founding node. We also observed a star-burst pattern of haplotype distribution for each clade where younger “satellite haplotypes” radiated from the founding haplotype, followed by diversification during multiple, independent population expansions. However, of the 74 different haplotypes observed for symbiotic genes, no single haplotype was found in all three continents. This is despite the fact that one haplotype shared between Chinese and North American populations were discovered. A highly unusual feature of the haplotype network was observed: Most links between sequential haplotypes consisted of multiple mutation steps and edges with single SNPs that were usually associated with the terminal nodes of the network. Since each SNP was associated with one single genetic event, the genetic dis-continuum between haplotypes and continents suggested long persistence of individual haplotypes and no recent wave of global transmission during the evolutionary history of symbiotic genes, although nearly all haplotypes represented by more than one sample were found in multiple locations in China. At the continental level, the North American population exhibited relatively high haplotype diversity, despite a small sampling size and being mostly located in Clade III. Only one large node was calculated for the German population, which was connected to North American and Chinese haplotypes, reflecting potential dominance of this symbiotic gene haplotype in Germany. By contrast, the Chinese population presented a much larger number of nodes in loosely defined clusters, representing greater haplotype diversity and a more complex population structure. At the species level, the distribution of haplotypes was almost unconstrained by species boundary. Linkage disequilibrium was tested for each STRUCTURE clade, and *I*_A_ tests revealed a strong recombination signature for symbiotic genes in Clade II (fig. 3).

### Demographic Trends

**Fig. 4.**
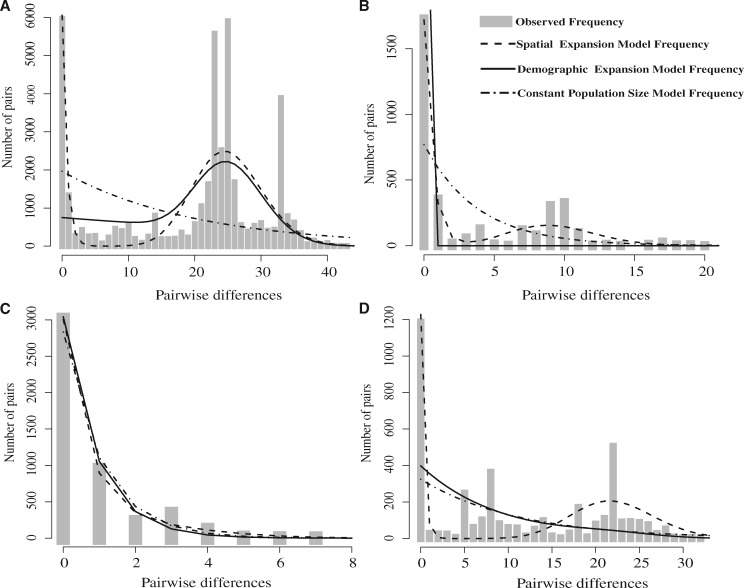
—Mismatch distributions of symbiotic genes. Frequency distributions of observed pairwise nucleotide differences in concatenated symbiotic genes based on multilocus sequence typing (gray bars), with expected distributions modeled under three different demographic scenarios (black lines). (*A*) All pairwise comparisons; (*B*) Clade I only; (*C*) Clade II only; and (*D*) Clade III only.

Signatures of past demographic events should be detected by the frequency distribution of pairwise nucleotide site differences in neutrally evolving loci. We performed mismatch distribution analysis in an attempt to obtain evidence of past demographic trends by comparing observed pairwise numbers of differences with distributions simulated under models of demographic expansion, spatial expansion, and constant population size (fig. 4). The mismatch distribution for all pairs of sequences fitted a bell-shaped curve with a mean of 19.6 mismatches (table 2) and a tail representing a high frequency of pairs with only a few mismatches (fig. 4*A*). The observed distribution did not depart significantly from the expected spatial expansion model (*H*_Rag_, *P *=* *0.411) but did differ significantly from the simulated distribution for either a demographic expansion or a stable population model (both *P *=* *0). The better fit to spatial expansion models was confirmed since the *SSD* was lower for the spatial expansion model (*SSD* = 0.029) than both the population expansion model and constant population size model (*SSD* = 0.047 and 0.064, respectively; table 2). For the whole data set, positive values of Tajima’s *D* and nonsignificance of Fu’s *F*_s_ values did not support an overall past population expansion event. These results were confirmed by LAMARC analysis that provided a population growth rate parameter *g* close to zero. The mismatch distribution parameter τ, the age of a modeled expansion event measured in mutational steps, was estimated at 23.6 mutational steps (95% CI, 16.78 − 29.50) under the assumption of spatial expansion.

**Table 2:** Summary Statistics of Mismatch Distribution Analyses and Neutrality Tests

	All	Clade I	Clade II	Clade III
Number of sequences	286	82	97	87
Number of haplotypes	64	17	17	21
Variable site (average number of pairwise differences)	111 (19.601)	35 (3.321)	18 (0.644)	75 (10.522)
Demographic expansion				
τ	25.65[16.232, 30.846]	0[0.00, 0.457]	3[0.342, 3.965]	23.05[0.429, 112.05]
Goodness-of-fit	0.047 (0.0)/0.062 (0.0)	0.265 (0.0)/0.194 (0.998)	0.004 (0.569)/0.223 (0.575)	0.092 (0.032)/0.142 (0.005)
Spatial expansion				
τ	23.580[16.784, 29.449]	8.933[0.134, 14.927]	2.53[0, 5.339]	21.886[14.197, 29.247]
Goodness-of-fit	0.0286 (0.333)/0.062 (0.411)	0.009 (0.812)/0.194 (0.737)	0.002 (0.623)/0.223 (0.631)	0.031 (0.844)/0.142 (0.75)
Fu and Li’s *F*_s_	−2.715 (0.351)	−3.349 (0.12)	−19.77 (0.0)	1.493 (0.719)
Tajima’s *D*	0.302 (0.621)	−1.668 (0.012)	−2.335 (0.001)	−0.968 (0.137)
*R* ^2^	0.0874 (0.679)	0.0456 (0.036)	0.0293 (0.0314)	0.0676 (0.173)
Growth rate (g)	−0.91[−284.08, 268.68]	7.3[−728.2, 2,329.07]	5,094[1,332, 10,000]	−4.14[−526.01, 474.71]

Note.—For both expansion models (sudden demographic expansion and special expansion), the results of tests of goodness-of-fit are provided (*SSD*/*H*_Rag_) with their significance. Growth rates were also calculated for each clade by LAMARC.

We next examined whether this pattern of expansion held true within each of the three genetically discontinuous clades when calculated individually. Evidence for spatial expansion was apparent in all three clades (fig. 4*B*–*D*), whereas Clade II was found to have undergone a population expansion (fig. 4*C*). The negative Tajima’s *D* and Fu’s *F*_s_ values also indicate demographic expansion in Clade II, and the LAMARC growth rate indicated that Clade I and Clade II grew while Clade III remained constant. However, despite the large positive estimate of *g* for Clade I, the CI was relatively large and included zero, hence it is quite likely that there was in fact little or no growth, as indicated by the mismatch distribution. The estimated times of expansion for the spatial expansion scenarios differed greatly for three clades; Clade III expanded earliest with 21.9 mutational steps, followed by Cluster I with 8.9 steps, and Clade II underwent the most recent expansion at 2.5 steps. In addition, the demographic expansion time for Clade II was close to its spatial expansion time (table 2). Taken together, these results provide evidence for the spatial expansion of symbiotic genes in all clades occurring at different times, assuming a varied evolutionary rate or pattern for the three clades.

### Phylogeographic Root of Symbiotic Genes

**Fig. 5.**
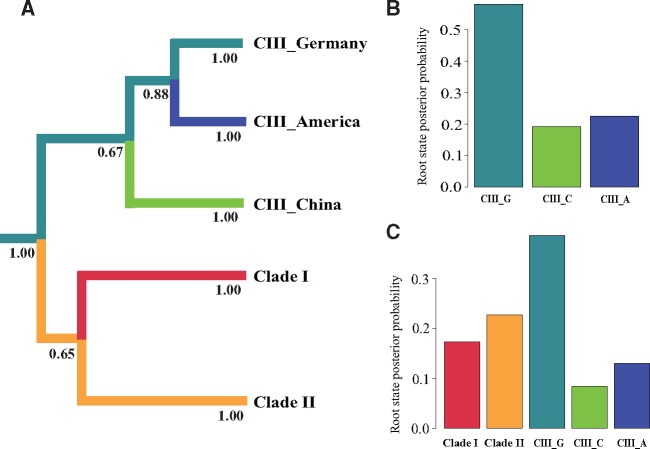
—Root state probabilities of symbiotic genes by clade and subclade inferred using BEAST. Branches of summarized maximum clade credibility phylogeny (*A*) for concatenated symbiotic genes based on multilocus sequence typing are colored according to the most probable location or clade state of their descendent nodes. Posterior probabilities of major branches supporting the tree topology are displayed below each branch. Root state posterior probabilities indicate that the German subclade is the most probable origin of all haplotypes (*B*) and Clade III symbiotic gene (*C*).

We conducted Bayesian multilocus phylogeographic analysis to infer the geographic location of the root. Root state reconstruction produced the highest posterior probabilities for Germany as the root state for both the whole data set and for Clade III symbiotic genes (fig. 5). For each locus independently, four of five loci supported the German subclade as the root (supplementary fig. S7, Supplementary Material online).

### Divergence Times

**Fig. 6.**
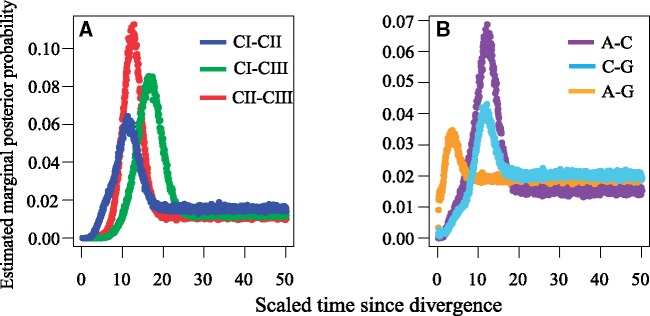
—Marginal posterior probability density distribution of relative divergence times in pairwise comparisons among clades (*A*) and among subclades in Clade III (*B*) of symbiotic genes estimated using IMa2. Smaller means and modes of the scaled time since divergence indicate more recent divergence of clades/subclades.

Divergence times were estimated between clades, and then between geographic subclades in Clade III (fig. 6). Clade I displayed the most ancient divergence from Clade III, and divergence between Clade I and Clade II was slightly earlier than that between Clade II and Clade III. Converting the mutation-scaled time estimates (*t*) to generations gave a splitting time for each pairwise analysis of *T*_CI__–__CII_ = 192,710, *T*_CI__–__CIII_ = 281,961, and *T*_CII__–__CIII_ = 219,745 generations before present (gbp). Posterior distributions had distinct but overlapping peaks for each pairwise analysis, and the tail hovered at a low level beyond *t* = 20, resulting in a broad highest posterior density interval. Therefore, obtaining smaller highest posterior density intervals may require more sequencing effort. In Clade III, CIII-Germany and CIII-North America exhibited much more recent divergence than if they diverged from CIII-China at almost the same time.

### Evolutionary Models of Symbiotic Genes

**Fig. 7.**
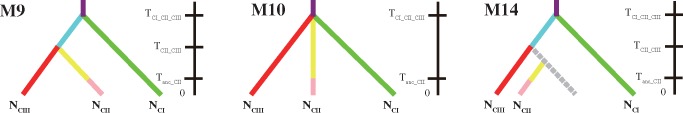
—Final models used to examine the relationships among clades of symbiotic genes. The three models are (*A*) simple divergence of clades such that Clade III is an ancestral lineage, (*B*) emergence of the three clades simultaneously, and (*C*) emergence of Clade II from an unsampled clade derived from Clade III. Model 10 is the most likely of the three models, with a posterior probability (PP) of 0.89.

We used ABC to reconstruct the demographic history of the three main clades of symbiotic genes. The ABC model choice procedure provided the strongest statistical support for Model 10 in comparison with other models. A very high relative posterior probability of 0.89 was obtained by Model 10, indicating that the three clades (N_CI_, N_CII_, and N_CIII_) diverged simultaneously (fig. 7). This result was confirmed by the high pairwise BF values obtained between Model 10 and all other scenarios (supplementary table S4, Supplementary Material online). The second-ranked model, Model 9, which assumed that Clade II split from Clade III after the divergence of Clade I and Clade III, received markedly smaller support than Model 10 and obtained a posterior probability of 0.09. The third best model, Model 14, in which an unsampled population derived from the ancestral population is the source of Clade II, had a posterior probability of 0.01.

We next checked whether the power of the analysis was sufficiently high to discriminate between the top three competing models. Type I and type II errors for Model 10 were estimated and 98.5% of data sets simulated under Model 10 produced the highest posterior probability for this model (type I error = 0.015). Pseudo-observed data sets generated under Models 9 and 14 were wrongly assigned to Model 10 at frequencies of 0.08 and 0.058, respectively (type II error), indicating the robustness of our model selection with a statistical power >90%. To assess the goodness-of-fit of the selected model to the data, we examined whether the selected model can reproduce the observed summary statistics by comparing the summary statistics estimated from the observed data and those obtained from the posterior predictive simulations. Almost all observed statistics fell well within the density distributions of the posterior predictive simulations for Model 10, indicating that the observed data were plausible under these models (supplementary fig. S3, Supplementary Material online). Moreover, the observed values mostly fell within the simulated data (*P *=* *0.18), suggesting that the assumed model was capable of reproducing the observed summary statistics.

To check whether the marginal posterior distributions estimated from the best models were biased, we generated 1,000 pseudo-observed data sets for the best model and tested the uniformity of posterior quantile distributions for each parameter. Validation of marginal posterior distribution by Kolmogorov–Smirnoff tests showed that most of the parameter estimates were unbiased with the exception of N_CI_, N_CII_, and N_CIII_ (supplementary fig. S8, Supplementary Material online). Although caution should be exercised when interpreting these parameters, visual inspection of the distributions of posterior quantiles indicated that most deviated only slightly from uniformity. Generally, this simulation-based evaluation of the performance of the model choice procedure showed that the ABC framework could reliably distinguish between the alternative divergent scenarios given the size and polymorphism of our data set, and provided clear and strong support for the simultaneous divergence of the three clades. Under Model 10, the estimated time of divergence among the three clades had a peak value of 204,300 (95% CI, 31,622 − 1,000,000) gbp (supplementary fig. S9, Supplementary Material online). This estimation coincided with that of IMa2. We also obtained evidence for a >100-fold demographic expansion in Clade II occurring 3,690 (95% CI, 251 − 79,433) gbp. The divergence between Chinese and North American subclades in Clade III was estimated to occur 16,900 gbp.

## Discussion

It has been suggested that a combination of geographic, ecological, and genetic factors has exerted strong influence on the genetic structure of rhizobial populations (Hirsch 1996). However, the association between legume host migration and symbiotic gene flow has received little attention. Herein, we tested whether the center of origin of one legume plant host coincides with that of its specific symbiotic genes. We chose black locust-rhizobia partners because this legume host is known to have undergone cross-continental migration during the last few centuries (Sabo 2000), and the symbiotic genes of black locust-associated rhizobia exhibit a compelling monophyletic origin compared with other legumes (Mierzwa et al. 2010). The multilocus approach outlined in the present study was inspired by the potential of MLST to accurately mirror the evolutionary relationships and trace the origins of symbiotic genes. Our choice of multilocus genotyping scheme allowed us to uncover hidden diversity and test assumptions regarding the distribution of genetic variation in symbiotic genes. This approach is especially suitable for shedding light on recent and deeper evolutionary relationships among symbiotic gene clades across the main cultivation areas of black locust.

### Genetic Diversity and Genetic Structure

Analysis of sequence variation revealed that the nucleotide diversity of all symbiotic genes was an order of magnitude lower than that of housekeeping genes. However, the genetic diversity of symbiotic genes was probably severely underestimated, given the probable host selection of specific sequence genotypes (Laguerre et al. 2003). Host specificity in rhizobia is determined by symbiotic genes, but whether/to what extent symbiotic genes suffer natural selection during interactions with legume remains unclear. Here, we performed neutrality test for each genes by the ratio of substitution rates at nonsynonymous and synonymous sites. But it should be noted that the *d*_N_/*d*_S_ ratio was originally developed for application to distantly diverged sequences, the differences among which represent substitutions that have fixed along independent lineages. Nevertheless, if the *d*_N_/*d*_S_ measure is applied to sequences sampled from close related individuals (i.e., within a single population, the differences among which represent segregating polymorphisms), *d*_N_/*d*_S_ is relatively insensitive to the selection coefficient, and *d*_N_/*d*_S_ < 1 can occur under both purifying and positive selections (Kryazhimskiy and Plotkin 2008). Here, our symbiotic genes represent a close related lineage with very low genetic variations, we cannot just interpret *d*_N_/*d*_S_ < 1 as purifying selection. We therefore applied the relative abundance of nonsynonymous and synonymous polymorphisms (*p*_N_ and *p*_S_) furthermore to measure the direct effect of natural selection in coding sequences (Stukenbrock and Bataillon 2012). Patterns of nonsynonymous and synonymous polymorphisms (*p*_N_ and *p*_S_) were analyzed jointly with rates of substitutions (*d*_N_ and *d*_S_) to assess the effect of natural selection oncoding sequences. Generally, *p*_N_/*p*_S_ > *d*_N_/*d*_S_ indicates purifying selection, *p*_N_/*p*_S_ < *d*_N_/*d*_S_ indicates positive selection and *p*_N_/*p*_S_ = *d*_N_/*d*_S_ indicates neutral evolution. We found that for symbiotic genes *p*_N_/*p*_S_ ≈ *d*_N_/*d*_S_ and *p*_N_/*p*_S_ > *d*_N_/*d*_S_ for housekeeping genes. These results rejected the hypothesis of positive selection for symbiotic genes during interactions with legume but provided strong evidences that most housekeeping genes were subject to purifying selection and the variation within these genes is nearly neutral, as suggested by most MLST schemes (Margos et al. 2008).

Recombination in the symbiotic genes was found to be very rare relative to mutation, indicating that symbiotic plasmid loci (Wang et al. 2018) are relatively clonal. By contrast, signals of recombination were found for the housekeeping genes, consistent with a previous report in *Sinorhizobium* and *Bradyrhizobium* (Tian, Young, et al. 2010).

Symbiotic gene transfer in rhizobia species was substantiated by misalignment of the genetic structure based on housekeeping and symbiotic genes, and the disorganized distribution of haplotypes of each species in the minimum spanning network. This finding is in agreement with previous research suggesting that the spread and maintenance of symbiotic genes occurred through vertical transmission, while horizontal transfer also played a significant role in rhizobia (Wernegreen and Riley 1999; Moulin et al. 2004; Chen et al. 2008; Tian, Chang, et al. 2010).

### Germany Is the Origin of Symbiotic Genes

We found multilocus support for a German origin of symbiotic genes associated with black locust. Bayesian phylogeographic analysis rooted both Clade III and all-clades to Germany for four of five symbiotic loci. STRUCTURE analysis of *M. huakuii* species revealed a consistent pattern of divergence between symbiotic and housekeeping genes, indicating little (if any) HGT with other genotypes and *M. huakuii* in Germany is probably the most ancient lineage harboring black locust-specific symbiotic genes. More than 80% of strains in Germany belong to the *M. huakuii* species, with clear divergence from the Chinese population. Moreover, we observed that the German haplotype dominated Clade III, where a pattern of sequence divergence corresponding to the continent appeared relatively recently in the evolutionary history of symbiotic genes, and no shared haplotypes were detected in all three continents, providing evidence of allopatric diversification. This strongly suggests that the three geographic subclades once belonged to a shared ancestral admixed population, but are now isolated.

However, due to sampling limitations in Europe and ancestral locations in the eastern United States, there is no doubt that our inference of phylogeographic origin hold true only for the regions where we collected strains. More sampling of strains from ancestral locations in the eastern United States is critical to trace the evolutionary trajectory of symbiotic genes. But it should be noted that previous studies on evolutionary history of symbiotic genes of *Robinia pseudoacacia* rhizobia (Wei et al. 2009; Mierzwa et al. 2010) as well as our phylogeny analysis with other legume species-associated symbiotic genes (results not shown) demonstrated that despite taxonomic diversity, *Robinia*-associated rhizobia from different continents share highly similar symbiotic genes and formed a branch of their own, suggesting that these symbiotic genes might have a monophyletic origin and a degree of host specificity. Thus *Mesorhizobium* symbionts sampled in Berkeley did not likely evolved from other California legumes associated rhizobia. In the absence of strains from ancestral locations, the evidence for an European origin for symbiotic genes is of particular interest in light of an Europe-to-China spread of *Robinia*. Our finding that the most ancestral genotype was found exclusively in Europe is compatible with the explanation that if *Robinia* first migrated across the Atlantic to the Europe in 1601 (Vitkova et al. 2017), it must be from the Appalachian mountain in the eastern United States, where *Robinia* originated. Thus European *Robinia* trees had the opportunity to hold the ancestral genotype of symbiotic genes. The divergence between China subclade and Gernany/America subclades in Clade III was estimated to occur 16,900 gbp (≈500 years ago, if we assumed a growth rate of 30 gen/year), which coincides with the timescale of introduction of *Robinia* to Europe. The split between Gernany and America subclade in Clade III was estimated more recently than they diverged from China subclade, concurring with the wide spread of *Robinia* in the United States one or two centuries ago. It is unknown how ancestral genotype in Europe might have been transported to the west coastal site of the United States, but the possibility that the same ancestral genotype residing in the eastern United States migrated to the western United States with its host cannot excluded. These findings may suggest that the spread of *Robinia* worldwide have contributed substantially to the distribution pattern of symbiotic genes in Clade III.

Given the slow evolution of rhizobia symbiotic genes compared with housekeeping genes, even a small amount of sequence divergence may take a long period of time to occur, long before the modern migration of black locust over the past few hundred years. If one haplotype emerged in one continent in recent centuries and went on to seed other continents since then, we would expect some of the same haplotype in all continents, and we would not have observed signatures of ancient range expansions in all continents. Therefore, our analysis provides phylogeographic evidence that the emergence of symbiotic genes in different continents is the result of multiple independent emergence events since ancient spreading from the origin center into its current range, and not a consequence of plant host migration.

It is not known how symbiotic genes were transported across continents before the migration of host plants, but dust storms may function as a vector for the transfer of bacterial cells (Vinuesa et al. 2005). As an assumed origin center, Germany would be expected to retain more than one haplotype since the divergence; it is not unlikely that with greater sampling we would have observed more haplotypes occurring in this continent. Indeed, Wei et al. (2009) have observed other haplotypes for *nodA*, *nodC*, and *nifH* genes in Germany. Notably, these were quite phylogenetically close to the present results observed in Germany, implying that these ancient haplotypes in Germany evolved slowly, or that the current introduction of black locust conferred a selective advantage on these haplotypes.

### Demographic History of Symbiotic Gene Clades

We observed a pattern of appearing relatively ancient sequence divergence that resulted in at least three major clades as revealed by IMa2 and ABC analyses, which also allows for strong quantitative inferences about the demographic history of symbiotic gene clades in our study areas. First, we estimated that the three clades split from each other 204,300 (95% CI, 31,622 − 1,000,000) gbp. Without a known average generation time for *Mesorhizobium* in natural populations, it is difficult to accurately pinpoint the time since the inferred divergence events. Nonetheless, *Mesorhizobium* is a slow-growing genus of bacteria and often undergoes differentiated events leading to the formation of bacteroids, the N_2_-fixing form of rhizobia in nodules; thus, its generation time is likely longer than most medically important bacterial taxa in natural populations. Denison and Kiers (2011) estimated that each rhizobial cell founding a population in soybean root nodules will generate an average of 10^8^ descendants in a few months, many more than if it had remained in the soil. This translates to an average of 27 generations for rhizobia in nodules per year. Considering the time *Mesorhizobium* spends during its life cycle reproducing in soil and dormant in nodules, and approximations of its doubling time observed under optimal conditions in the laboratory (Moukoumi et al. 2013), we assumed that *Mesorhizobium* could go through a maximum of 30 generations per year in natural environments. Accordingly, the time since divergence of symbiotic genes in the main clades is at least several thousand years, long before the modern cross-continental movement of black locust.

A genetic signature of spatial expansion and population expansion in Clade II was reflected in the sequence mismatch distributions, which also allowed for estimation of the timescale of ancient expansion events. The timing of expansion events measured in number of mutational steps (τ) was approximately three under both supported expansion scenarios. This parameter is related to time *t* since expansion according to the formula τ = 2μ*t*, where μ is the total mutation rate per generation per gene. ABC analysis estimated that the mutation rate spans ∼10^−8^ (95% CI, 0.5 × 10^−8^−2 × 10^−8^) per nucleotide per generation. Therefore, the point estimate from this expansion time was *∼*24,695 (95% CI, 4,939 − 98,781) gbp, consistent with the ABC analysis prediction that the expansion event most likely occurred 3,690 (95% CI, 251 − 79,433) gbp. Given the generation time spanning 30 generation per year as above, the time since expansion in Clade II indicated by the mismatch distribution parameters spans 122 − 822 years before present. This estimation of the age of expansion events in Clade II coincides with the timescale of introduction and spreading of black locust in China (Ren et al. 2014).

The average number of pairwise differences in Clade II was only 0.644 substitutions (table 2), much lower than other clades and the 19.6 mismatches that we used to infer the timescale of the major prehistoric expansion event for the whole symbiotic genes data set, indicating a relatively young clade. These findings probably suggest that a stronger selective advantage of symbiotic genes in Clade II compared with other clades drives strains to form nodules with black locust in China.

### Phylogeographic Pattern in China

By grouping the haplotypes into three main clonal clades with inferred founders, we discovered clues about the origins and directionality of ancient spreading of symbiotic genes in our study areas. The founding haplotypes in both Clade I and Clade II (i.e., both clades distributed mainly in China, except for two haplotypes of Clade I found in North America) were not restricted to one or a few close sites, indicating no distinct phylogeographic pattern in China, and the two clades of symbiotic genes in China have multiple isolated, independent centers of origin. We would not expect such high levels of diversity to be present if these symbiotic genes had colonized China only recently; these results therefore support the hypothesis that symbiotic genes have had a long association with China. It seems that the introduction of black locust did not introduce too many new symbiotic gene haplotypes. This finding further supports the scenario that the current distribution of genetic diversity of symbiotic genes is the result of independent emergence events of symbiotic gene clades in different continents, rather than being driven by host migration in the last few centuries.

Finally, the admixed clade observed in STRUCTURE analysis suggests limited contemporary recombination of symbiotic genes among clades. The recombination mainly occurred in China, where our sampling was extensive enough to capture some signals of recombination between haplotypes. The *I*_A_ index also supports recombination of symbiotic genes within Clade II, indicating the wide prevalence of Clade II in China. This finding is consistent with the observation of expansion of Clade II in China based on mismatch distribution analysis.

This study provides information on the evolution pattern of symbiotic genes in black locust*-*associated rhizobia. Our results demonstrate that the current distribution of genetic diversity in different continents resulted from independent, isolated ancient demographic processes, including spatial expansions occurring at least on the order of thousands of years ago, and divergence between continents, but not recent plant migration. Continental isolation of symbiotic gene genotypes may have agricultural implications due to differences in the nodulation ability of rhizobia associated with specific genotypes. Sequencing more rhizobial strains from broader locations around the world and detailing the evolutionary history of black locust will provide further insight into coevolution between rhizobia and this legume.

## Supplementary Material

Supplementary data are available at *Genome Biology and Evolution* online.

## Supplementary Material

Supplementary_Material_evz116

## Notes

**Data deposition:** The sequences of 286 *Mesorhizobium* strains have been deposited in the GenBank under accession numbers MH780206–MH780491 and MH785495–MH788636.
