# Supplementary material for: New Insight into the Evolution of Symbiotic Genes in Black Locust-Associated Rhizobia
Source: Genome Biol Evol. 2019 Jun 13;11(7):1736–50. doi: 10.1093/gbe/evz116 (PMC6698633; doi:10.1093/gbe/evz116)
Supplement: Supplementary_Material_evz116 [file supplementary_material_evz116.doc]

**Supplementary material for**

**New insight into the evolution of symbiotic genes in black locust*-*associated rhizobia**

Zhenshan Liu^1^, Weimin Chen^1*^, Shuo Jiao^1^, Xinye Wang^1^, Miaochun Fan^1^, Entao Wang^2^ and Gehong Wei^1*^

**^1^** State Key Laboratory of Crop Stress Biology in Arid Areas, Shaanxi Key Laboratory of Agricultural and Environmental Microbiology, College of Life Sciences, Northwest A&F University, Yangling, Shaanxi 712100, China;

^2^ Departamento de Microbiología, Escuela Nacional de Ciencias Biológicas, Instituto Politécnico Nacional, 11340 México, D.F., Mexico

**^*^**Correspondence: Gehong Wei, College of Life Sciences, Northwest A&F University, Yangling, Shaanxi 712100, China.

Tel.: +86-02987080009; E-mail: weigehong@nwsuaf.edu.cn

Weimin Chen, College of Life Sciences, Northwest A&F University, Yangling, Shaanxi 712100, China.

Tel.: +86-02987080009; E-mail: chenwm029@nwafu.edu.cn

**Table S1** Sampling information for 286 rhizobial strains tested in this study

| Code | Species | Country | Code | Species | Country |
| --- | --- | --- | --- | --- | --- |
| B15 | *M. ciceri* | North America | HZ55 | *M. amorphae* | China |
| B17 | *M. ciceri* | North America | HZ56 | *M. amorphae* | China |
| B19 | *M. amorphae* | North America | HZ57 | *M. amorphae* | China |
| B2 | *M. huakuii* | North America | HZ58 | *M. amorphae* | China |
| B20 | *M. ciceri* | North America | HZ59 | *M. loti* | China |
| B24 | *M. ciceri* | North America | HZ6 | *M. loti* | China |
| B25 | *M. ciceri* | North America | HZ61 | *M. amorphae* | China |
| B26 | *M. ciceri* | North America | HZ63 | *M. qingshengii* | China |
| B29 | *M. ciceri* | North America | HZ7 | *M. amorphae* | China |
| B30 | *M. amorphae* | North America | HZ8 | *M. amorphae* | China |
| B32 | *M. ciceri* | North America | HZ9 | *M. amorphae* | China |
| CCNWGS0123 | *M. amorphae* | China | JJ17 | *M. amorphae* | China |
| CCNWSX0028 | *M. loti* | China | JN32 | *M. amorphae* | China |
| CCNWSX0079 | *M. amorphae* | China | JN33 | *M. metallidurans* | China |
| CCNWSX0125 | *M. amorphae* | China | KY12 | *M. loti* | China |
| CCNWSX0132 | *M. loti* | China | KY13 | *M. metallidurans* | China |
| CCNWSX0144 | *M. amorphae* | China | KY6 | *M. loti* | China |
| CCNWSX0192 | *M. qingshengii* | China | KY8 | *M. loti* | China |
| CCNWYC115 | *M. robiniae* | China | KY9 | *M. metallidurans* | China |
| G20 | *M. amorphae* | China | N100 | *M. amorphae* | China |
| G22 | *M. amorphae* | China | N101 | *M. amorphae* | China |
| G27 | *M. qingshengii* | China | N116 | *M. amorphae* | China |
| G37 | *M. amorphae* | China | N117 | *M. amorphae* | China |
| G7 | *M. qingshengii* | China | N118 | *M. amorphae* | China |
| Ger11 | *M. loti* | Germany | N119 | *M. amorphae* | China |
| Ger12 | *M. huakuii* | Germany | N121 | *M. sangaii* | China |
| Ger13 | *M. huakuii* | Germany | N125 | *M. huakuii* | China |
| Ger15 | *M. huakuii* | Germany | N127 | *M. amorphae* | China |
| Ger16 | *M. sangaii* | Germany | N129 | *M. amorphae* | China |
| Ger17 | *M. huakuii* | Germany | N133 | *M. amorphae* | China |
| Ger19 | *M. huakuii* | Germany | N136 | *M. huakuii* | China |
| Ger2 | *M. huakuii* | Germany | N138 | *M. amorphae* | China |
| Ger21 | *M. huakuii* | Germany | N139 | *M. amorphae* | China |
| Ger22 | *M. huakuii* | Germany | N140 | *M. amorphae* | China |
| Ger23 | *M. huakuii* | Germany | N141 | *M. amorphae* | China |
| Ger24 | *M. huakuii* | Germany | N143 | *M. amorphae* | China |
| Ger26 | *M. loti* | Germany | N144 | *M. amorphae* | China |
| Ger27 | *M. loti* | Germany | N145 | *M. amorphae* | China |
| Ger28 | *M. huakuii* | Germany | N148 | *M. amorphae* | China |
| Ger29 | *M. huakuii* | Germany | N150 | *M. amorphae* | China |
| Ger3 | *M. huakuii* | Germany | N151 | *M. amorphae* | China |
| Ger30 | *M. huakuii* | Germany | N154 | *M. amorphae* | China |
| Ger31 | *M. huakuii* | Germany | N155 | *M. amorphae* | China |
| Ger32 | *M. huakuii* | Germany | N156 | *M. amorphae* | China |
| Ger33 | *M. huakuii* | Germany | N157 | *M. amorphae* | China |
| Ger34 | *M. huakuii* | Germany | N158 | *M. amorphae* | China |
| Ger35 | *M. huakuii* | Germany | N162 | *M. amorphae* | China |
| Ger37 | *M. huakuii* | Germany | N166 | *M. amorphae* | China |
| Ger38 | *M. huakuii* | Germany | N167 | *M. huakuii* | China |
| Ger39 | *M. loti* | Germany | N168 | *M. huakuii* | China |
| Ger4 | *M. huakuii* | Germany | N19 | *M. amorphae* | China |
| Ger40 | *M. huakuii* | Germany | N32 | *M. amorphae* | China |
| Ger41 | *M. huakuii* | Germany | N37 | *M. amorphae* | China |
| Ger42 | *M. huakuii* | Germany | N38 | *M. amorphae* | China |
| Ger43 | *M. huakuii* | Germany | N4 | *M. amorphae* | China |
| Ger47 | *M. huakuii* | Germany | N51 | *M. huakuii* | China |
| Ger48 | *M. huakuii* | Germany | N58 | *M. amorphae* | China |
| Ger5 | *M. huakuii* | Germany | N64 | *M. ciceri* | China |
| Ger6 | *M. huakuii* | Germany | N7 | *M. amorphae* | China |
| Ger7 | *M. huakuii* | Germany | N70 | *M. huakuii* | China |
| Ger8 | *M. huakuii* | Germany | N71 | *M. amorphae* | China |
| Ger9 | *M. loti* | Germany | N80 | *M. qingshengii* | China |
| GX1 | *M. huakuii* | China | N85 | *M. amorphae* | China |
| GX10 | *M. loti* | China | N86 | *M. amorphae* | China |
| GX12 | *M. loti* | China | N87 | *M. amorphae* | China |
| GX16 | *M. amorphae* | China | N88 | *M. amorphae* | China |
| GX19 | *M. loti* | China | N89 | *M. amorphae* | China |
| GX2 | *M. amorphae* | China | N90 | *M. amorphae* | China |
| GX21 | *M. loti* | China | N92 | *M. amorphae* | China |
| GX22 | *M. loti* | China | N93 | *M. amorphae* | China |
| GX23 | *M. loti* | China | N94 | *M. amorphae* | China |
| GX24 | *M. amorphae* | China | N95 | *M. amorphae* | China |
| GX25 | *M. loti* | China | N96 | *M. amorphae* | China |
| GX26 | *M. loti* | China | N97 | *M. amorphae* | China |
| GX27 | *M. metallidurans* | China | N98 | *M. amorphae* | China |
| GX28 | *M. amorphae* | China | N99 | *M. amorphae* | China |
| GX29 | *M. amorphae* | China | P1T11 | *M. huakuii* | Germany |
| GX30 | *M. amorphae* | China | P2T13 | *M. sangaii* | Germany |
| GX31 | *M. loti* | China | P3T23 | *M. loti* | Germany |
| GX32 | *M. amorphae* | China | QD2 | *M. huakuii* | China |
| GX34 | *M. loti* | China | QD6 | *M. huakuii* | China |
| GX35 | *M. amorphae* | China | QD9 | *M. huakuii* | China |
| GX37 | *M. amorphae* | China | SM13 | *M. amorphae* | China |
| GX38 | *M. metallidurans* | China | T14 | *M. metallidurans* | China |
| GX40 | *M. loti* | China | T20 | *M. amorphae* | China |
| GX41 | *M. amorphae* | China | T30 | *M. metallidurans* | China |
| GX42 | *M. amorphae* | China | T36 | *M. qingshengii* | China |
| GX43 | *M. amorphae* | China | T37 | *M. metallidurans* | China |
| GX44 | *M. amorphae* | China | T42 | *M. metallidurans* | China |
| GX45 | *M. amorphae* | China | T44 | *M. metallidurans* | China |
| GX47 | *M. loti* | China | T45 | *M. amorphae* | China |
| GX48 | *M. amorphae* | China | T51 | *M. metallidurans* | China |
| GX49 | *M. amorphae* | China | T52 | *M. amorphae* | China |
| GX52 | *M. amorphae* | China | T56 | *M. amorphae* | China |
| GX53 | *M. amorphae* | China | T63 | *M. metallidurans* | China |
| GX8 | *M. loti* | China | T64 | *M. amorphae* | China |
| HL1 | *M. huakuii* | China | TH13 | *M. metallidurans* | China |
| HL11 | *M. huakuii* | China | TH2 | *M. huakuii* | China |
| HL16 | *M. huakuii* | China | TH20 | *M. loti* | China |
| HL17 | *M. huakuii* | China | TH24 | *M. qingshengii* | China |
| HL18 | *M. huakuii* | China | TH25 | *M. qingshengii* | China |
| HL19 | *M. huakuii* | China | TH26 | *M. qingshengii* | China |
| HL2 | *M. huakuii* | China | TH28 | *M. metallidurans* | China |
| HL21 | *M. amorphae* | China | TH30 | *M. sangaii* | China |
| HL9 | *M. huakuii* | China | WF1 | *M. huakuii* | China |
| HZ11 | *M. amorphae* | China | WF7 | *M. huakuii* | China |
| HZ12 | *M. amorphae* | China | WF8 | *M. huakuii* | China |
| HZ13 | *M. amorphae* | China | WH11 | *M. loti* | China |
| HZ14 | *M. amorphae* | China | WH12 | *M. amorphae* | China |
| HZ15 | *M. amorphae* | China | WH16 | *M. amorphae* | China |
| HZ16 | *M. amorphae* | China | WH17 | *M. sangaii* | China |
| HZ17 | *M. amorphae* | China | WH26 | *M. amorphae* | China |
| HZ19 | *M. amorphae* | China | WH27 | *M. amorphae* | China |
| HZ2 | *M. amorphae* | China | WH28 | *M. amorphae* | China |
| HZ21 | *M. amorphae* | China | WH29 | *M. amorphae* | China |
| HZ22 | *M. amorphae* | China | WH30 | *M. amorphae* | China |
| HZ23 | *M. amorphae* | China | WH31 | *M. amorphae* | China |
| HZ24 | *M. huakuii* | China | WH35 | *M. amorphae* | China |
| HZ26 | *M. huakuii* | China | WH37 | *M. huakuii* | China |
| HZ28 | *M. huakuii* | China | XY1 | *M. loti* | China |
| HZ29 | *M. huakuii* | China | XY21 | *M. loti* | China |
| HZ3 | *M. amorphae* | China | XY22 | *M. loti* | China |
| HZ3_3 | *M. qingshengii* | China | XY27 | *M. loti* | China |
| HZ30 | *M. huakuii* | China | XY28 | *M. loti* | China |
| HZ31 | *M. amorphae* | China | XZ1 | *M. amorphae* | China |
| HZ32 | *M. huakuii* | China | XZ2 | *M. amorphae* | China |
| HZ33 | *M. huakuii* | China | XZ3 | *M. amorphae* | China |
| HZ35 | *M. amorphae* | China | XZ4 | *M. amorphae* | China |
| HZ36 | *M. amorphae* | China | XZ5 | *M. amorphae* | China |
| HZ37 | *M. loti* | China | XZ6 | *M. amorphae* | China |
| HZ39 | *M. amorphae* | China | XZ7 | *M. amorphae* | China |
| HZ4 | *M. amorphae* | China | XZ8 | *M. amorphae* | China |
| HZ4_4 | *M. amorphae* | China | YH1 | *M. metallidurans* | China |
| HZ40 | *M. amorphae* | China | YH10 | *M. metallidurans* | China |
| HZ41 | *M. huakuii* | China | YH11 | *M. metallidurans* | China |
| HZ42 | *M. amorphae* | China | YH12 | *M. amorphae* | China |
| HZ43 | *M. amorphae* | China | YH13 | *M. amorphae* | China |
| HZ44 | *M. loti* | China | YH14 | *M. metallidurans* | China |
| HZ45 | *M. loti* | China | YH15 | *M. amorphae* | China |
| HZ46 | *M. loti* | China | YH8 | *M. metallidurans* | China |
| HZ47 | *M. amorphae* | China | YH9 | *M. metallidurans* | China |
| HZ49 | *M. amorphae* | China | Rob10 | *M. sangaii* | Germany |
| HZ5 | *M. amorphae* | China | Rob11 | *M. sangaii* | Germany |
| HZ50 | *M. amorphae* | China | Rob12 | *M. sangaii* | Germany |
| HZ51 | *M. amorphae* | China | Rob13 | *M. loti* | Germany |
| HZ53 | *M. amorphae* | China |  |  |  |

**Table S2** Substitution model, molecular clock and clock rates obtained in this study for each locus

| Locus | Substitution model | Clock | Clock rate | UCLD.stdev |
| --- | --- | --- | --- | --- |
| *nifA* | JC69 | Strict | 8.29E-03 | 0.22 |
| *nifH* | JC69+I | Lognormal | 2.51E-02 | 1.492 |
| *nodA* | JC69 | Strict | 6.01E-04 | 0.195 |
| *nodC* | JC69 | Strict | 7.36E-03 | 0.372 |
| *nolT* | JC69 | Strict | 1.09E-02 | 0.193 |

Abbreviations: UCLD.stdev, the standard deviation of the uncorrelated lognormal relaxed molecular clock.

**Table S3** Prior distributions for scenarios in ABCtoolbox analyses

| Parameter | Shape | Lower bound | Upper bound |
| --- | --- | --- | --- |
| N_CI_ | Log-uniform | 2 | 5.7 |
| N_CII_ | Log-uniform | 2 | 5.4 |
| N_CIII_ | Log-uniform | 2 | 6 |
| Nanc_CII | Log-uniform | 2 | 5 |
| NCI_CII | Log-uniform | 2 | 6 |
| NCI_CIII | Log-uniform | 2 | 6 |
| NCII_CIII | Log-uniform | 2 | 6 |
| NCI_CII_CIII | Log-uniform | 2 | 6 |
| Tanc_CII | Log-uniform | 2 | 6.6 |
| TCI_CII | Log-uniform | 2 | 6.6 |
| TCI_CIII | Log-uniform | 2 | 6.6 |
| TCII_CIII | Log-uniform | 2 | 6.6 |
| TCI_CII_CIII | Log-uniform | 2 | 6.7 |
| Mutation rate | Log-uniform | -10 | -7 |
| Proportion from parent population | Uniform | 0.001 | 0.999 |

Abbreviations: N_CI_, N_CII_, and N_CIII_, effective populations of Clades I, II, and III, respectively.

**Table S4** Approximate Bayesian computation model choice based on Bayes factor and posterior probability.

| Model | *P*-value | *PP* | *BF* |
| --- | --- | --- | --- |
| M1 | 0.0296 | 7.63×10^-8^ | 2.57×10^-5^ |
| M2 | 0.1836 | 1.16×10^-3^ | 1.16×10^-3^ |
| M3 | 0.1018 | 3.46×10^-5^ | 6.14×10^-3^ |
| M4 | 0.0126 | 5.99×10^-8^ | 3.31×10^-5^ |
| M5 | 0.1934 | 1.48×10^-3^ | 1.49×10^-3^ |
| M6 | 0 | 5.55×10^-35^ | 1.10×10^-30^ |
| M7 | 0.0002 | 2.74×10^-34^ | 3.05×10^-30^ |
| M8 | 0.1484 | 3.07×10^-3^ | 3.07×10^-3^ |
| M9 | 0.2906 | 9.08×10^-2^ | 9.99×10^-2^ |
| M10 | 0.1808 | 0.887 | 7.878 |
| M11 | 0.0406 | 3.00×10^-11^ | 1.02×10^-7^ |
| M12 | 0.0346 | 3.51×10^-10^ | 3.35×10^-7^ |
| M13 | 0.16 | 6.28×10^-6^ | 1.51×10^-3^ |
| M14 | 0.2284 | 1.31×10^-2^ | 1.33×10^-2^ |
| M15 | 0.1316 | 2.03×10^-4^ | 9.33×10^-3^ |
| M16 | 0.1968 | 1.18×10^-3^ | 1.18×10^-3^ |
| M17 | 0.1188 | 7.54×10^-4^ | 6.70×10^-3^ |
| M18 | 0.2362 | 7.65×10^-4^ | 7.65×10^-4^ |
| M19 | 0.1342 | 3.95×10^-5^ | 4.54×10^-3^ |

Abbreviations: *P*-value, the fraction of retained simulations with the marginal likelihood smaller or equal to that of the observed data; *PP*, posterior probability; and *BF*, Bayes factor.


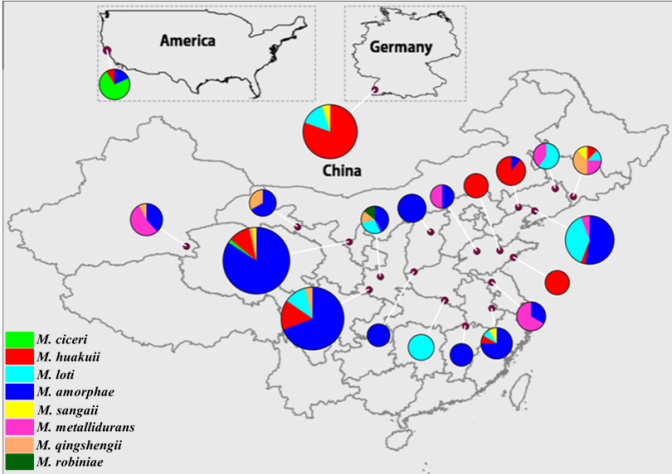

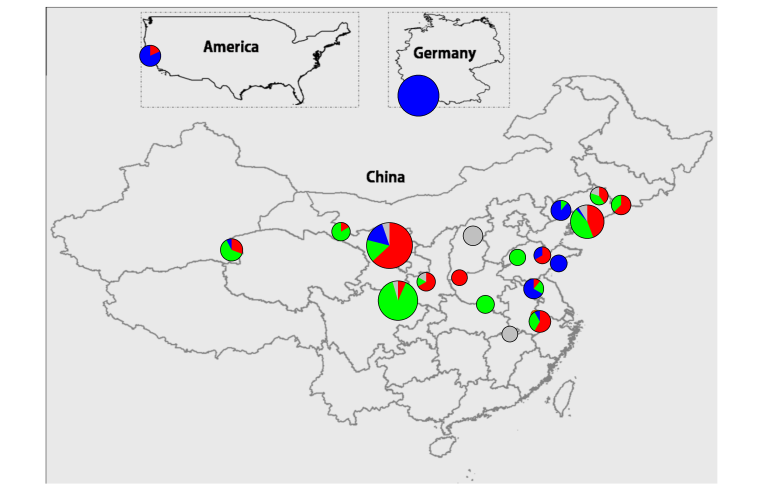


**Clade I**

**Admixed clade**

**Clade II**

**Clade III**

A

B

**Fig. S1.** Species (A) and clade (B) distributions of the 286 *Mesorhizobium* strains associated with black locust in the sampling areas.


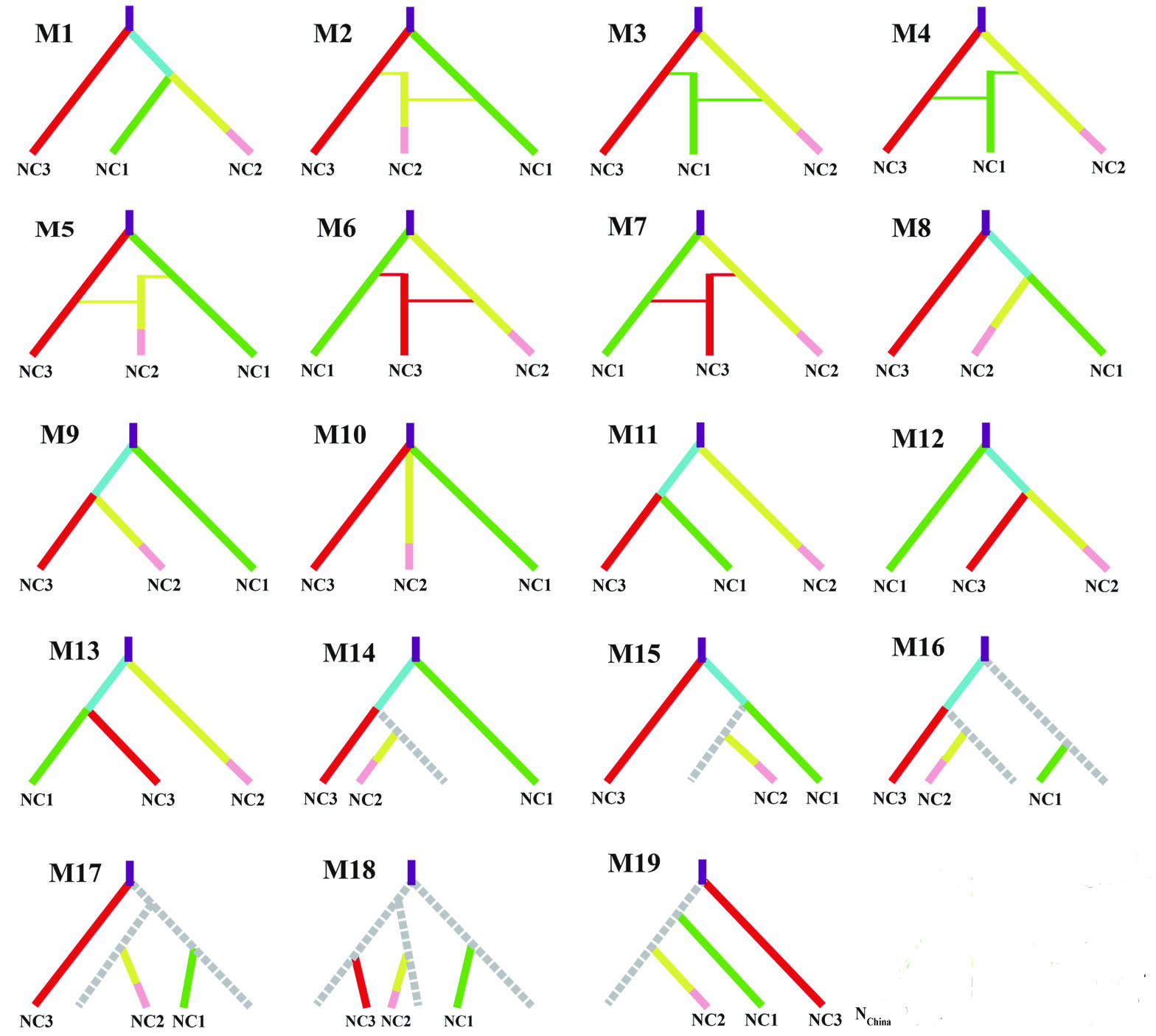


**Fig. S2.** Scenarios used to test evolutionary relationships of symbiotic genes among clades using ABCtoolbox. Present-day populations are at the base of the schematic tree. Ancestral relationships among these populations are represented by lines intersecting in the past, with the vertex of the schematic tree representing the most recent common ancestor of all samples.

**
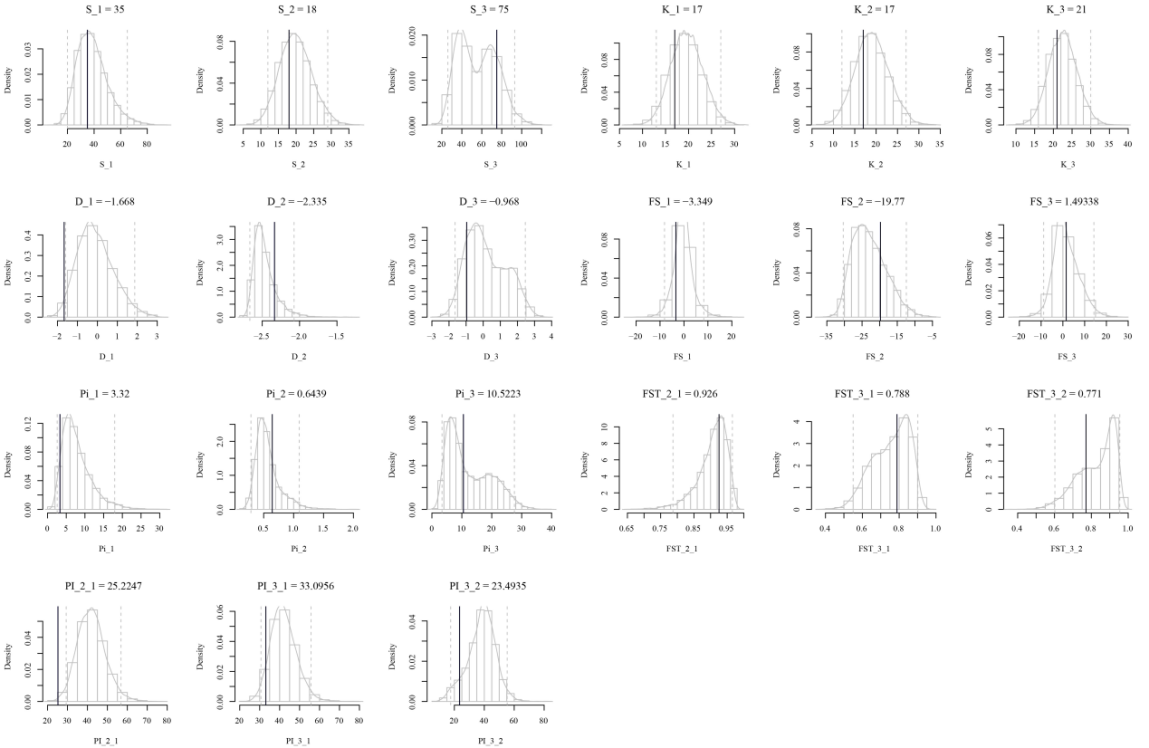
**

**Fig. S3.** Density distributions of 21 summary statistics (histogram) with 2.5% and 97.5% quantiles (dashed vertical lines) describing genetic diversity of symbiotic genes within and among clades. Distributions were generated from the 10,000 simulations closest to the observed dataset of scenario (ii). Corresponding observed summary statistics for each plot are shown in black.


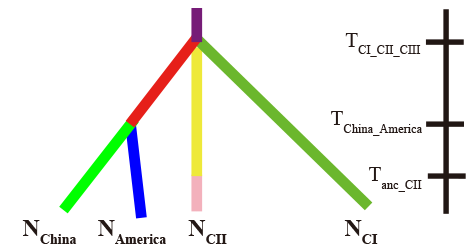


**Fig. S4.** The best scenario used to examine the relationships among clades and between Chinese and North American geographic subclades in Clade III by approximate Bayesian computation. The German subclade was excluded from this analysis due to its lack of sequence variation.

**
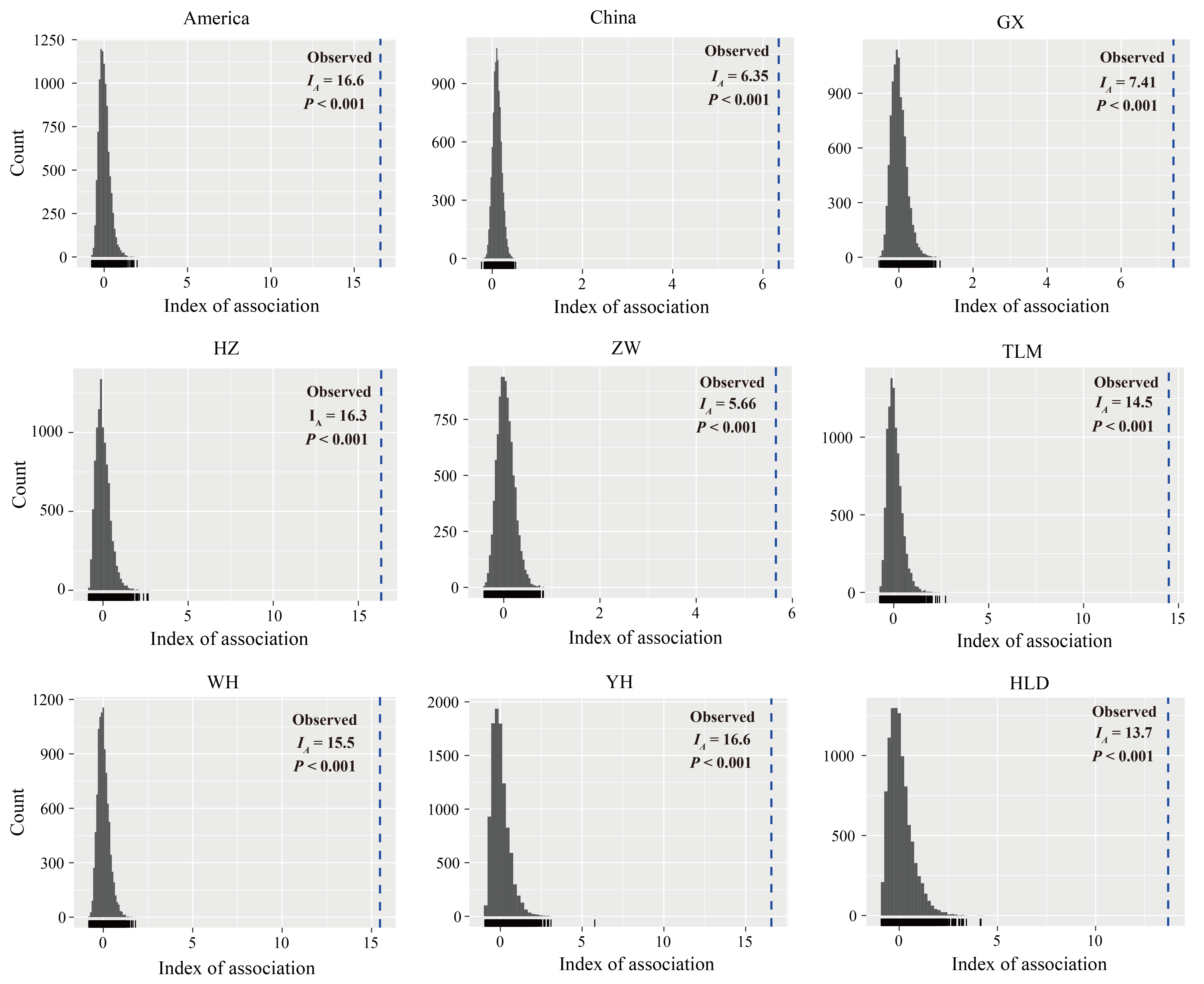
**

**Fig. S5.** Index of association (*I_A_*) test of recombination for each sampling site with number of individuals > 9 as well as China as a whole.

**
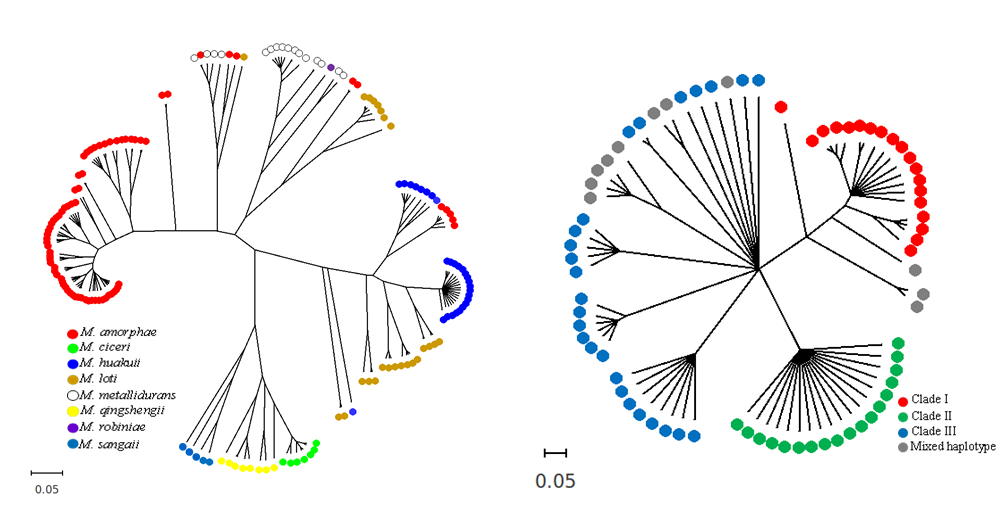
**

**Fig. S6.** Majority-rule consensus tree from concatenated sequences of housekeeping gene haplotypes (A) and symbiotic gene haplotypes (B) based on the posterior distribution of genealogies inferred by ClonaFrame.

**
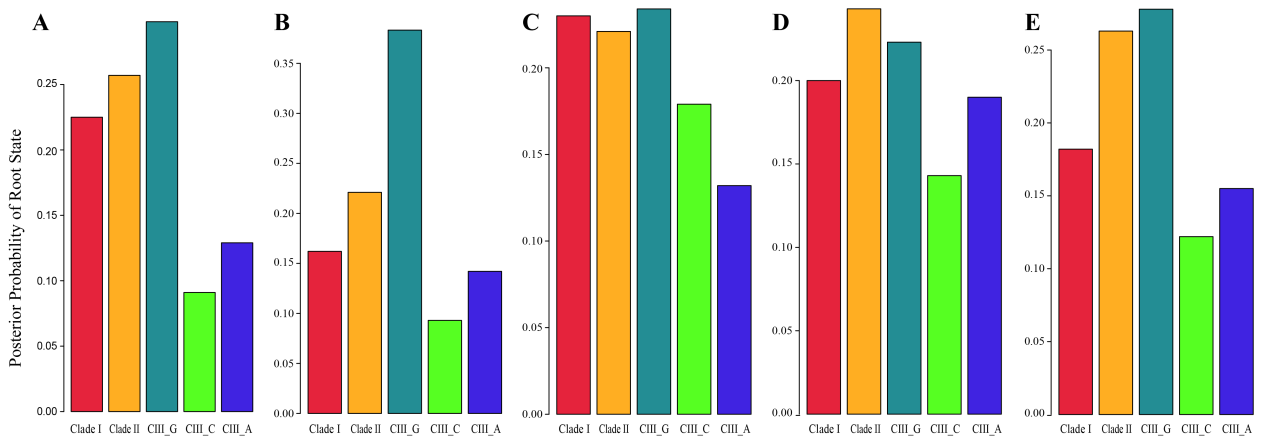
**

**Fig. S7.** Root state posterior probabilities for each clade/subclade of symbiotic genes. Independent analysis of each locus resulted in the German subclade having the highest probability for the root. (A) *nifA*; (B) *nifH*; (C) *nodA*; (D) *nodC*; and (E) *nolT*.

**
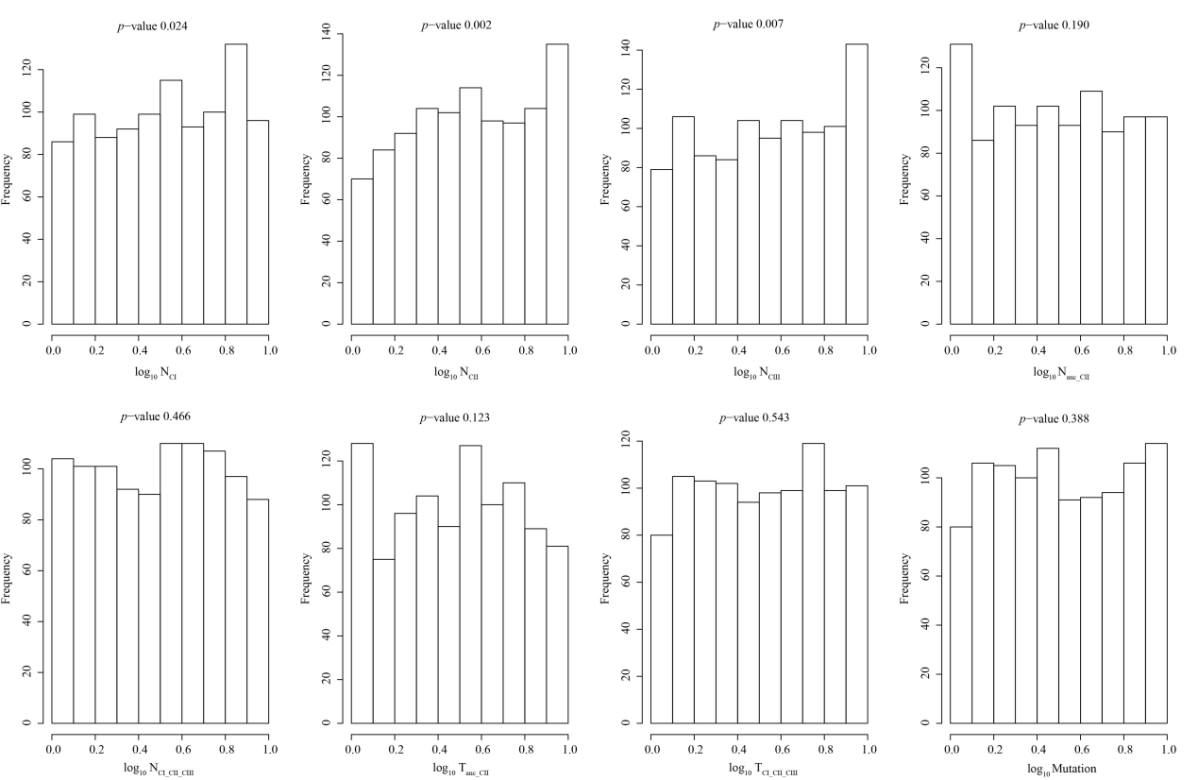
**

**Fig. S8.** Distribution of posterior quantiles of parameters for the most probable evolutionary model of symbiotic genes, for evaluating potential bias in parameter estimates measured by a departure from a uniform distribution using Kolmogorov-Smirnov tests. Analyses are based on 1,000 pseudo-observations.

**
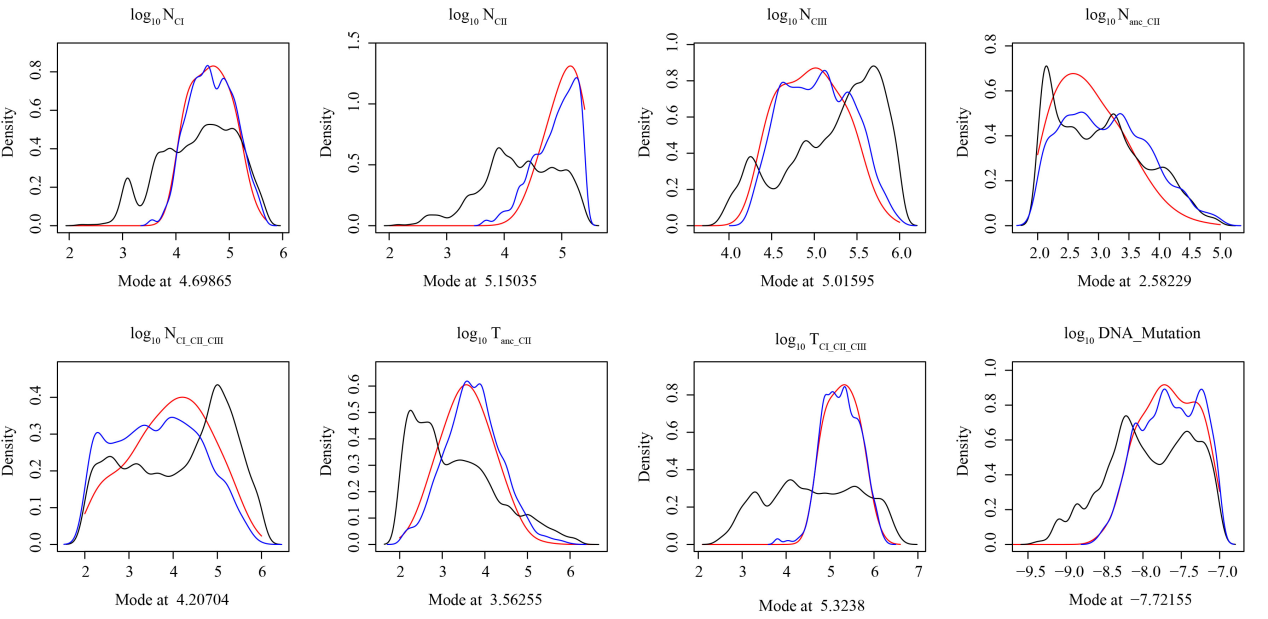
**

**Fig. S9.** Posterior (red), marginal (blue), and prior (black) distributions of demographic parameters for the best scenario of symbiotic genes, plotted using the 5,000 closest simulations to the observed dataset.
